# Macroscopic and Microscopic Thermodynamics: From Fundamentals to Present Applications, 2nd Edition

**DOI:** 10.3390/ijms25168618

**Published:** 2024-08-07

**Authors:** Ana M. Mainar, José S. Urieta

**Affiliations:** 1 Group of Applied Thermodynamics and Surfaces (GATHERS), Aragon Institute for Engineering Research (I3A), Universidad de Zaragoza, 50018 Zaragoza, Spain; 2 Departamento de Química Física, Facultad de Ciencias, Universidad de Zaragoza, 50009 Zaragoza, Spain

## 1. Some General Points

This Special Issue, “*Macroscopic and Microscopic Thermodynamics: From Fundamentals to Present Applications. 2.0*” was proposed with the aim of highlighting some of the most important and contemporary aspects that the very broad field of thermodynamics offers, both as a doctrine and as a tool for a very wide range of scientific disciplines, including technical and industrial applications.

Since its initial development as a mature science throughout the 19th century, on the basis that thermodynamics primarily concerns topics relating to the interconversion of heat and other forms of energy, both its conceptual field and its use as a tool have expanded greatly. Indeed, throughout its two centuries of history, thermodynamics has acquired a leading reputation due to the style of its own developments and also because of its capacity as a very useful instrument with great potential to facilitate the development of many scientific fields that are relatively closely related to physics and beyond [1,2]. The applications of thermodynamics in cosmology [3], information and computer science [4], and even social sciences such as economic systems [5,6], and traffic flow [7], are examples of the latter.

As is widely acknowledged, the most classical thermodynamic studies, i.e., those corresponding to *macroscopic thermodynamics*, are capable of making accurate and valuable predictions about processes involving an enormous variety of systems without taking into account their intimate structures. Nevertheless, moving beyond the last century, a new qualitative advance has emerged. This advance was made possible thanks to an adequate molecular vision of systems and particularly thanks to the developments in *statistical mechanics*. The aim of this formalism is to explain the physical properties of matter in bulk based on the dynamical behavior of its microscopic constituents. For this purpose, statistics and mechanics are used; subsequently, from that microscopic vision, the thermodynamic properties of a significant variety of systems in equilibrium, or even out of equilibrium, can be deduced. This molecular approach, corresponding to *microscopic thermodynamics*, is especially essential in the developments of chemical thermodynamics and chemical engineering. In general, statistics mechanics is divided into two forms: (i) the form dealing with systems in thermodynamic equilibrium, that is, historical statistical thermodynamics, and (ii) the form dedicated to the study of transport phenomena (the transport of matter, heat, etc.) and chemical reactions, that is, non-equilibrium statistical mechanics. Within these contexts, and in general scientific terminology, the phrase *chemical thermodynamics* refers to studies interrelating energy with chemical reactions or with a physical change of state within the frame of the laws of thermodynamics. In turn, Prausnitz [8] defines *molecular thermodynamics* as a synthesis of classical and statistical thermodynamics, molecular physics, and physical chemistry capable of providing quantitative information on the equilibrium properties of a variety of fluid mixtures. This process is essential in the development of chemical engineering. Conversely, for systems that are changing or may change over time and that are subject continuously or discontinuously to a flow of matter or energy to or from other systems, the theoretical treatments applied to equilibrium need to be implemented, including the laws of evolution regarding the changes of the state variables with time.

It is also remarkable that in recent decades, thanks to the increase in computational calculation capacity, the prediction and correlation of thermodynamic quantities through empirical, semi-empirical, and molecular models have made it possible to find solutions to many practical problems for simple systems or complex mixtures. Thus, the term *computational thermodynamics* has been applied to the use of computers to solve problems, ranging from typically scientific problems, such as those of thermodynamics in materials science [9], to those already mentioned in sociological areas.

With the purpose of systematizing and simplifying the above nomenclature and concepts, thermodynamics conceived as a discipline is usually divided into several fundamental branches, according to the different conceptual models considered, their theoretical or experimental basis, and the different types of systems under study. When addressing these bases, three branches are frequently considered: (*i*) *classical thermodynamics*, which corresponds to what we refer to as *macroscopic thermodynamics*, (*ii*) *statistical thermodynamics*, and (*iii*) *chemical thermodynamics* (including equilibrium thermodynamics and non-equilibrium thermodynamics). The last two branches, (*ii*) and (*iii*), correspond to what we refer to in the present Special Issue as *microscopic thermodynamics*.

When approaching thermodynamic methods as a tool for interpreting the behavior of numerous systems, we find a considerable number of fields in which thermodynamics has and will continue to find very important applications. In fact, thermodynamics has been penetrating the borders of many classical and current scientific fields. It covers broad and important aspects within the developments of engineering [10] (including, for example, chemical engineering [11], engineering biosystems [12], and food processing [13]), electrochemistry [14], energy use and conversion [15], materials science [16], biology [17], vital processes and life science [18], nutrition [19,20], agriculture [21], geology [22], and also in modern aspects of architecture [23], cosmology (the thermodynamics of solar systems [24], black holes [25], and the thermodynamics of space-time [26,27]), the environment [28], sustainability [29], or the economy, ecology and society (ecosystem ecology [30], thermoeconomics and industrial ecology [31], and the thermodynamics of quality [32]).

Given the enormous variety of topics in which thermodynamics can play a leading role, the need to limit the number of topics to be discussed in a Special Issue (SI) such as the one to which this Editorial is dedicated, is obvious. With this purpose of self-limitation and the aim of achieving a certain level of coherence, a series of topics were chosen. These topics represent, bring together, and promote current lines of research, building bridges between the basic aspects of thermodynamic science and new developments in technological processes and materials.

Under the umbrella defined by the proposed scopes, different types of systems, processes, and approaches of interest can be seen in the following SI. A set of nine research articles and two reviews are included, on which some brief comments are made and can be read below.

## 2. Overview of the Published Articles

The article by Gimeno et al. (Contribution 1) focuses on the thermodynamic behavior of a binary mixture (2-propanol + 1,8-cineole). The mixture is of particular interest both from a theoretical point of view and in light of its potential applications. These factors are corroborated by a detailed bibliographic review performed by the authors. Particularly noteworthy are the current and future applications of cineole in the field of pharmacy and medicine, in addition to the potential use of their mixtures with alcohols as additives for diesel or gasoline [33]. The authors describe the experimental in study of vapor–liquid equilibrium (VLE) performed. The study is complemented by measurements of densities and enthalpies of mixing for the aforementioned system, in addition to adequate theoretical examination of the resulting data. The experimental results correspond, as a whole, to the temperature interval between 278.15 and 323.15 K. By using the VLE data, activity coefficients and excess Gibbs energies were calculated, and the thermodynamic consistency test between excess molar Gibbs energies and excess molar enthalpies was substantiated. VLE data were correlated via Robinson-Mathias and Peng-Robinson-Stryjek-Vera equations of state, both including the Peneloux volume translation correction. These data were also correlated using the SAFT model. This latter approach offers a molecular vision quite suitable for systems with highly non-spherical or associated molecules. Of the three theoretical approaches considered, SAFT is the one that best approximates the volumetric behavior for the present system. Regarding the VLE, the three models provide theoretical values reasonably close to the experimental ones. Such results allow the performance of these approximations to be positively evaluated for the type of mixtures such as the one considered here. From a different perspective, when comparing the actual system with that resulting in the replacement of the cyclic ether with the corresponding lineal ether, a relatively higher value of the OH–O interaction energy between molecules of cineol and propanol can be deduced. This fact was interpreted by the authors based on the 3D structure of cineol and propanol molecules and based on an increase in the electron density of the oxygen atom in the cyclic molecule. Lastly, from the various conclusions derived from this work, it can be highlighted that the study provides suitable data and updates on developments for the simulation and design of separation processes to obtain chemical substances of interest, such as 1,8-cineole. In addition, from a microscopic perspective, the work provides new bases for the elucidation of the interaction forces between molecules carrying ether or alcohol functional groups.

The authors of Contribution 2, Semenov et al., report precise data on one of the non-variant equilibrium four phases of the binary system (CO_2_ + H_2_O). In relation to the simple p-T phase diagram of carbon dioxide, the introduction of the second component, water, significantly complicates the aforementioned diagram, even if extreme conditions are not considered. The above is particularly due to the appearance of a clathrate hydrate, a carbon dioxide hydrate, that is, a crystalline inclusion compound. This type of compound, clathrate hydrates, is an ice-like material and consists of a solid network of hydrogen-bonded water molecules creating cavities capable of enclosing “guest” molecules, such as the considered carbon dioxide or other non-polar compounds, such as some noble gases, methane, or small hydrocarbon chains [34]. The existence of a liquid phase rich in supercooled water is also considered in this work. Due to the existence of the number of different phases, a series of quadruplet points can appear, for some of which the corresponding values of pressure and temperature have been determined previously and can be found in the literature [35]. Two of these points, Q1 and Q2, fall within pressure and temperature ranges not far from ambient conditions. In their contribution to the present SI, the authors highlight the notable interest in the physicochemical properties of clathrate hydrates and their numerous current and potential applications. Areas of application include seawater desalination, the separation of gas mixtures, environmental preservation, energy storage, and food industry technologies. The work by Semenov et al. is of particular relevance regarding these applications. Because of this interest and in order to gain a better understanding of various processes in which the CO_2_–water system plays an important role, precise knowledge of the singular points in its phase diagram is remarkably beneficial. Based on this premise, the authors formulated a new technique for the direct determination of the P and T coordinates of the lower quadruple point corresponding to the equilibrium between the gas–water solution, ice, and gas hydrate. In their study, they demonstrated the satisfactory reproducibility of their results, adequate reduction in measurement time, and a significant reduction in the combined standard uncertainty of the equilibrium temperature. This reduction in uncertainty is by an order of magnitude when compared with that of the previous data in the literature. Thus, the applied technique, which is described in great detail by the authors, is of great interest in validating similar techniques applicable to other systems.

Contribution 3 by Golikova et al. focuses, in particular, on the study of the excess enthalpies of the binary mixtures involved in the quaternary mixture acetic acid + n-butanol + n-butyl acetate + water. This four-component mixture is the most characteristic in the formation of butyl ester from the corresponding acid and alcohol. It should be noted that although it is a reactive mixture and the reaction processes are equilibrium type, for the reaction to proceed at an appreciable speed, it must be heated and homogeneous (e.g., strong acids) or heterogeneous catalysts must be present. In fact, butyl acetate has been classically manufactured via the Fischer esterification of butanol and acetic acid in the presence of sulfuric acid, which is used as a catalyst [36]. It should be noted that butyl acetate is an important chemical in high demand. The butyl acetate market represents about 2 billion USD annually. Because of its low volatility, its most common industrial use is in the production of lacquers and paints as a solvent. It is also applied in the manufacturing of artificial leather, plastic resins, and adhesives, as an anticorrosive agent, and for hardened coatings. In the pharmaceutical industry, it is used as a solvent or as an extraction agent. Additionally, it is applied in several cosmetic products such as lacquers, perfumes, or oils, in addition to nail polish removers. Thus, it is of interest to learn in detail all of the technical particularities that may be involved in the synthesis of the ester. In the contribution by Golikova et al., a detailed review of the mixing enthalpies of binary systems between the mentioned four components is carried out, highlighting the gaps or inconsistencies between the corresponding data for these mixtures. In order to address the main shortcoming mentioned above, the authors carried out experimental measurements, at 313.15 K and atmospheric pressure, of the excess enthalpies of three of the six possible binary mixtures between the four components. They include the binary mixtures acetic acid + n-butanol, acetic acid + n-butyl acetate, and n-butanol + n-butyl acetate. To this end, they used effective experimental equipment, namely a Setaram C80 isothermal mixing calorimeter. Their experimental results were correlated through the NRTL model and the Redlich–Kister equation. Using the obtained results and data from the literature, other thermodynamic magnitudes ($C_{p,m}^{E}$, $S_{m}^{E}$, Δ_*mix*_*S*_*m*_, $G_{m}^{E}$, and Δ_*mix*_G_*m*_) of the binary systems were estimated. The value and significance of the results obtained relate not only to their technical interest in relation to the industrial manufacturing of butyl acetate ester; there is also undouble theoretical interest derived from the close relationship between the magnitude studied experimentally (enthalpies of excess) and the interaction between the molecules that constitute the systems, among others. This type of interaction is a hot topic within studies focused on physical chemistry and other related sciences.

In Contribution 4 by Carbone et al., the authors discuss the surface tension of a series of binary mixtures with special characteristics. Surface tension, usually described as the tendency of liquid surfaces at rest shrink to the minimum surface area possible, is an important factor in industrial and technical processes; however, it is also of great theoretical significance in the scientific field. This is because surface tension is also directly related to intermolecular forces in the system. The binary mixtures considered by the authors consist primarily of 1,2 dibromoethane with a series of hydrocarbons (c-C6, n- and i-C7, n-C8, n-C14), coupled with 1-chloronaphthalene + 2,2,4-trimethylpentane, 1,4-dioxane +cyclohexane and methanol+methyl-tertbutyl-ether, with the latter selected as a reference. The primary aim of the work was to elucidate whether systems with W-shaped $C_{p}^{E}$ vs. composition curves also show an anomalous dependence of surface tension on concentration. The authors rely on the hypothesis that the strong concentration fluctuations typical of systems with W-shaped $C_{p}^{E}$ curves could also manifest themselves in the surface properties. It should be noted that, as reported by Saint-Victor and Patersoni [37], in the 1980s, Grolier, Wilhelm, and their collaborators found many systems with a “W-shape” for the $C_{p}^{E}$ vs. composition curves. These systems were mainly constituted by an alkane plus a chemically dissimilar compound (1,4-dioxane, 2-butanone and 3-pentanone, 1,2-dichloroethane, 1,1,2,2-tetrachloroethane, 1,4-dichlorobutane, 1,6-dichlorohexane, and others). Although some authors initially suggested that this peculiar dependence could be a consequence of “a component existing as conformers of different polarity, the relative proportions of which are changed through mixing with the other component”, the aforementioned authors, Saint-Victor and Paterson, although agreeing that such changes may be important, proposed another possibility. They pointed out that the W-shape may be due to deviation in local from bulk composition. Then, based on the quasi-chemical lattice theory of solutions of Guggenheim, a qualitative interpretation can be made. According to the theory, random mixing induces negative $C_{p}^{E}$, whereas local non-randomness (concentration fluctuations) contributes positive values of $C_{p}^{E}$, with the W-shape resulting as a consequence of a greater dominance of concentration fluctuations at intermediate compositions versus the tendency toward a random nature at both ends of the composition range [38]. The authors of Contribution 4 carried out surface tension measurements by using three different procedures and completed their laboratory study with depolarized light scattering experiments to determine the concentration fluctuations. Among the other results, an anomalous concentration dependence for the surface tension and the mixing surface tension for the systems under study was reported. In addition, the liquid/vapor interface is proposed to have a high thickness for the mixtures, as a consequence of the high values of the concentration–concentration correlation function associated with strong concentration fluctuations.

Contribution 5 by Gallego et al. refers to the study of the eutectic mixture of urea and sodium acetate trihydrate. Within the context of the aim of sustainable natural resource management, this mixture is proposed as a potential suitable solvent for the extraction of the valuable compound collagen from by-products of industrial fish and aquaculture processing. From this perspective, the solid–liquid equilibrium of the aforementioned eutectic mixture was studied. Subsequently, the solubility of the main amino acids of fish collagen in the proper eutectic at different temperatures, and also in the mixture resulting from the dilution of this mixture with water (50 wt %), was determined. The authors highlight in their work the importance of better management of the hitherto poorly valued by-products generated in the worldwide capture fisheries and aquaculture production, representing in some cases up to 70% of the total mass processed. For the purposes of economic calculations, it can be stated that the current global average consumption of fish and other forms of seafood per person is roughly 20 Kg/year, which represents an enormous amount of by-products. These by-products comprise heads, skin, fish ends, viscera, scales, bones, or cartilage. Such by-products have been traditionally used, following simple processing, for the production of fishmeal or animal feed, fish liver oil, glue, gelatin, or fertilizer, or they can be simply discarded as waste. The latter option implies a direct impact on the environment, in addition to significant economic losses. Therefore, adequate treatment of by-products and waste is essential for their revaluation, since they offer valuable sources of bioactive compounds. The authors provide new data on the composition of the eutectic mixture of sodium acetate trihydrate + urea. As they suggest in their work, its temperature close to ambient temperature and the fairly high values found for the solubilities of the most abundant amino acids in collagen and fish gelatin make this eutectic mixture promising as a solvent for corresponding industrial extraction processes. Furthermore, the authors found that the incorporation of water into the eutectic mixture increases the solubility and allows the operating temperature to be lowered, which expands its scope. The authors also suggest future areas of study, including molecular simulation, in order to gain a better understanding of how amino acids are solvated in the eutectic medium.

In Contribution 6, Nikolić et al. present their BioMThermDB 1.0 database, which is a freely available, web-based database that reports on the thermophysical and dynamic properties of an extensive series of proteins, predominantly globular proteins and antibodies, in aqueous solutions. Those properties, including the hydrodynamic radius, electrophoretic mobility, zeta potential, and the cloud-point temperature, are of crucial importance when determining the stability of this type of compound. Their database systematizes information dispersed in bibliographies and complements other existing databases in order to resolve questions about phase stability and molecular interactions in the aqueous solution or address the influence of changes in concentration, temperature, or pH on the properties of said solutions. In relation to the *hydrodynamic radius*, it can be stated that it is one of several parameters that describe molecular size, that is, how big or small a molecule is. It is defined as the radius of a sphere with the same hydrodynamic properties (frictional coefficient) as the molecule, and it is measurable, for example, through the use of dynamic light scattering [39]. This parameter provides, for example, a simple way to confirm the identity and oligomeric state of a protein in a solution. Another one of the parameters considered, *electrophoretic mobility*, can be highlighted as a key parameter characterizing the motion of a charged particle in an electric field in free solution. This electrophoretic mobility is defined as the speed of movement of the species under an electric field with the value of unity. It can be measured through the use of a moving-boundary method or, alternatively, via microelectrophoresis, that is, through the measurement of the time taken for single particles to travel a defined distance. Although it is widely acknowledged that this mobility depends primarily on the size and net charge of the molecule, a detailed relationship is still lacking for molecules with complexity in the shape and charge distribution of proteins [40]. As such, it should be stated that both proteins and other biomolecules may be separated according to their varying electrophoretic mobility, making electrophoresis a rapid, simple, and sensitive analytical tool for this purpose. For its part, the *zeta potential* (ζ) is defined as the electric potential on the sliding surface that separates the thin liquid layer attached to the solvated protein particle when this moves, with respect to the rest of the liquid mass. It describes the magnitude of electrostatic interactions of the particles in solution and, therefore, is a determinant of the stability of the dispersion. Usually, the zeta potential is measured indirectly through the electrophoretic mobility of the particles. Values of this potential are particularly useful in the design of drugs that maintain their function while suspended in solution. Regarding the *self-diffusion coefficient*, the IUPAC definition states that it is nothing but the diffusion coefficient when the chemical potential gradient equals zero. It is determined by the random, thermal motion of the molecules in the absence of a concentration gradient and is experimentally obtained by using isotopic markers or through the pulsed-field gradient-NMR method. The self-diffusion coefficient provides information not only on the size, shape, or molecular weight of a species but also on its aggregation or association with other species and changes in structure, or denaturation; however, quantitative interpretation of diffusion coefficient data can be difficult [41]. The *viscosity* of protein solution has also been determined to elicit specific information about these compounds. For example, the intrinsic viscosity (the ratio of the increase in the relative viscosity by the solute to its concentration in the limit of infinite dilution) has been used as a measure of “denaturation” extent. Conversely, the viscosity control of concentrated protein solutions is vital for the manufacturability and drug delivery routes of many therapeutic proteins developed by the pharmaceutical industry [42]. Lastly, the *cloud-point temperature*, defined as the temperature where the protein solution undergoes phase separation into two co-existing phases, is indicative of protein interactions and constitutes a useful tool for food product engineering [43]. For precise experimental determination, an array of optical detectors continuously monitors the sample to detect the first appearance of a cloud of the new phase. In summary, the BioMThermDB 1.0 database presents an overview of thermodynamic and thermophysical data on protein solutions that should aid scientists in solving problems such as predicting protein structures, studying protein–protein interactions, and use in protein family identification and other theoretical treatments. In addition, as indicated by the authors, the database can be used to help researchers in planning new experiments on these systems.

Contribution 7, by Pekar, refers to systems kinetically governed by the simultaneous existence of reaction processes and matter transport by diffusion. Chemical reactions involve the material transformation of chemical substances over time until reaching a final equilibrium state; diffusion, in contrast, is a process in which chemical species spread out as a result of existing concentration gradients. As indicated by the author of the work, the simultaneous occurrence of chemical reactions and diffusion is commonly present in engineering processes and in the biological processes of living systems such as metabolism or respiration. Key examples can also typically be found in geological and environmental systems. Although the analysis of the reaction and diffusion processes can be performed from a microscopic perspective, through atomic theory in the first case (collision theory and transition state theory) and through statistical treatment in the latter case (as the type carried out in the deduction of the Einstein–Smoluchowski equation), a macroscopic, phenomenological approach (as are that of formal kinetics and that of Fick equations) is also perfectly possible. In the present contribution, it is precisely this latter approach, the macroscopic one, which is exclusively addressed. The developed work specifically targets the named self-balancing diffusion, as defined by Truesdell [44]. Thus, based on general relations for exchanges of mass among the constituents of a mixture, it is considered that the interpretation of the mass changes that occur in the physicochemical processes does not strictly require the terms “atom” and “molecule”, nor is a strict reference to “chemical” changes required. The only aspect that was necessary was to assume that “each constituent is made up by combination in fixed proportions from certain individually indestructible constituents”. In turn, in the corresponding stoichiometric developments, Truesdell followed the results presented by Bowen [45] for their developments in the theory of fields of chemical reactions and their equilibria. For his part, in the present contribution, the author addresses in detail the applicability of the concept of reaction extent (the extent to which the reaction has proceeded) to those defined as self-balancing diffusion processes, and, in general terms, he shows how they could be detected in reality. The author also addresses the practical manifestations and consequences of these types of processes.

Contribution 8 by Volodin et al. has as its primary aim the goal of deepening knowledge at a microscopic level of the fascinating compound octamethylcyclotetrasiloxane, whose study, at that particular level, presents some gaps in the current literature. This chemical is the most important of the cyclic siloxanes, with production of well over one hundred million kilograms/year, and it is of significant industrial interest, especially in the production of polymers and lubricants or as a finishing agent or solvent. As with any other chemical, knowledge of this compound at a molecular level is important not only from the perspective of basic knowledge but also because of the close relationship between its structure and its macroscopic properties. The latter are what ultimately determine the applications of the product. Therefore, structure–property and structure–function relationships have long been considered important explanatory and crosscutting concepts in the disciplines of chemistry and biology [46] and also in engineering and materials science. Indeed, a detailed understanding of the relationship between structure, function, and reactivity is essential for the success of modern science. In the work by Alexander et al., experimental X-ray diffraction and thermochemical measurements are presented. Complemented by their theoretical calculations (density functional theory, ab initio molecular dynamics, and metadynamics), the authors were able to thoroughly elucidate the structure of the solid phases of the compound below its melting point up to 100 K. They also determined the values of the energy barriers associated with the phase and conformational changes occurring in their molecules. In relation to these changes in the 3D molecular structure mentioned above, it can be stated that, for theoretical chemists, conformational isomerism is a form of stereoisomerism in which the isomers can be interconverted simply by rotations around formally single bonds. If a conformation corresponds to a local minimum on the energy surface, then these stereoisomers are specifically referred to as conformational isomers or conformers. Conversion of one conformer to another involves overcoming an energy barrier. If this barrier is low, the rotations occur freely and quickly and then a sample of a given chemical consists of a mixture of several conformers. Conversely, if the height of the energy barrier is sufficiently high, rotation will thus be restricted, and a molecule may exist for a relatively long time period as a stable rotamer. As noted by Shklover and Struchkov [47], the rings in organocyclosiloxanes (RR’SiO)n with n > 3 (the same constitution as the compound considered in Contribution 8) are non-planar, and hence, it may be expected that they exist in different conformations. The single-crystal X-ray diffraction technique utilized by the authors is a powerful tool capable of providing detailed information on the type of crystal structure, including information such as distances and bond angles. In the present case, the X-ray results form a basis for discussion that was combined with thermodynamic parameters obtained through DSC experiments and modern computational methods (DFT, molecular dynamics and metadynamics calculations). This process allowed the authors, among other objectives, to establish energy parameters and the pathway of phase transition, in addition to the establishment of the conformational changes associated with the observed phase transition.

In the paper by Le Carpentier et al. (Contribution 9), the authors report on research work in the field of cardiac muscle tissue. The authors of the paper respond to an interest in improving the understanding of heart physiology from all points of view, including the most fundamental aspects, such as that concerning physical science. This particular field of work, heart physiology [48], is in continuous evolution, in search of more effective treatments for cardiovascular conditions. Specifically, through the above study, the authors analyze the effect of cardiac tissue anoxia on the molecular mechanism of myocardial function from a thermodynamic perspective. Cardiac muscle tissue, known as the myocardium, is the striated muscle tissue that forms most of the wall of the heart. It is specialized in its continuous and involuntary contraction, which allows blood to be pumped through blood vessels to supply oxygen and nutrients to the entire body. The myocardium contains cells called cardiomyocytes. They possess a cylindrical, elongated shape and connect to one another, forming myocardial fibers. These fibers are separated by collagen which supports the capillary network of the tissue. The junctions between myocytes consist of special formations called intercalated discs that allow the rapid propagation of electrical impulses, ensuring the synchronized contraction of the cells, which generate the heartbeat. Most of the cytoplasm of cardiomyocytes is occupied by myofibrils. Myofibrils are composed of aligned sarcomeres that constitute the contraction units. In turn, these sarcomeres are composed of thin filaments of actin and thicker filaments of myosin, extended between the so-called “Z discs”. The muscular contraction of the myocardium occurs when the sarcomeres shorten owing to the fact that the filaments of these actin and myosin proteins slide over each other, making the Z discs move closer. Similar to many other aspects of biology [49], human cardiac activity has come to the attention of researchers from different scientific areas. As an example, Uehara and Sakane [50] utilized the thermodynamic approach in order to derive a relation between the referenced cardiac output (a global blood flow parameter of interest in hemodynamics) and the oxygen consumption rate. Shortly after their study, Dini et al. [51] examined the thermodynamics of metabolism and cardiac efficiency, including the entropy generation associated with the inefficiencies of the cardiac cycle. The experimental section of the work by Le Carpentier et al. is based on the observation of the effect of anoxia on the ventricular papillary muscles of adult male rats. Fundamentally, the shortening velocity of the aforementioned muscles was measured, and the associated isometric and isotonic tensions were determined. The subsequent theoretical treatment included the concepts of tribology corresponding to the friction of the myosin filaments, the rate of entropy production and excess entropy production associated with non-equilibrium, self-organization, and dissipative structures. The conclusions reached by the authors are of particular interest in relation to heart transplants.

In the review by Dehghan Niestanak and Unsworth (Contribution 10), the most relevant up-to-date knowledge on the interactions between blood proteins and specific uremic toxins (UTs) present in the human bloodstream when individuals are affected by certain conditions, are reviewed. When kidney function declines, reaching a value below a certain critical value, clinical alterations derived from the accumulation of UTs begin to manifest, which poses a serious risk to health. UTs have been defined as solutes normally excreted by the kidneys but retained in chronic kidney disease and interacting negatively with biological functions. They can be molecules derived from the intake of nutrients or products of metabolism itself. Roughly a quarter of the nearly 150 uremic retention solutes listed in 2012 by EUTox (a working group of the European Society of Artificial Organs) correspond to the so-called *Protein-Bound Uremic Toxins* (PBUTs). Although the molecular weight of most of these molecules is less than 500, they are difficult to remove as a result of their high affinity with plasma proteins. Of these molecules, the authors focused their attention on human serum albumin (HSA) in particular due to its relevance and abundance in the human bloodstream. The interactions between PBUTs and plasma proteins alter the conformation of the proteins, blocking other important unions with endogenous or exogenous substances that are necessary in order to maintain health. In addition, those undesirable unions with PBUTs reduce the effectiveness of palliative treatments. As a consequence of the aforementioned interactions, PBUTs are present in the bloodstream in two forms: a main bound fraction (bound to albumin and non-diffusible) and a small free fraction. From the numerous investigations focused on the elimination of UTs, it has been shown that the techniques to eliminate their free fraction are not very clinically effective due to their low proportion. For this reason, a considerable amount of research on the issue of patients with chronic kidney disease has been largely directed toward the use of so-called “chemical displacers” that could compete with PBUTs to bind to serum albumin [52]. The authors of the aforementioned review highlight the need to investigate in greater depth the molecular interactions that exist between the different species involved: PBUTs, serum albumin, and competitive binders. The authors discuss in detail the role of competitive binders in inhibiting uremic toxins. In summary, they carefully review the accuracy of critical information suitable for developing new pathways to remove undesirable uremic toxins from the blood compartment.

The review by Nie et al., corresponding to Contribution 11, represents a contribution to the scientific field of separation processes. These types of operations are frequently necessary in order to obtain pure substances or concentrates for domestic and industrial purposes or in research works. Chemical and engineering separations include a series of methods such as membrane techniques, distillation, crystallization, adsorption, absorption and stripping, and extraction. Specifically, liquid–liquid extraction is a technique involving two immiscible liquid phases. In this arrangement, one of the two phases, the solvent phase, extracts the solutes from the other liquid phase; however, this separation process must be followed by solvent recovery in addition to raffinate cleanup. Subsequently, because there are many options available to choose the fluid phases, for the raffinate phase cleaning method, and especially when we refer to industrial production, an adequate separation design is vital. This design must take into account factors such as efficiency, raw materials, energy cost, and also personnel and environmental safety. The work of the aforementioned authors refers to a specific type of liquid–liquid extraction, that is, the aqueous two-phase system (ATPS), and more specifically, the ionic liquid (IL)-based aqueous two-phase system (ATPS). This technique has attracted considerable attention in recent years as it includes the advantages of ILs and those of the ATPS. Such a combination represents an alternative that may be advantageous in certain cases compared to other types of ATPSs with other phase-forming chemicals [53] (polymer–polymer, polymer–salt, alcohol–salt, and micelles). In one instance, the ILs, usually defined as compounds that consist of an organic cation paired with an organic or inorganic anion with a melting point below 100 °C [54], constitute materials in which interest has grown exponentially in recent decades. They can compete successfully over conventional organic solvents, which are often more volatile and consequently more toxic, flammable, and environmentally harmful. Today, more than 137,066 results from the Web of Science Core Collection appear for the topic of *ionic liquid*. A significant portion of these works are devoted to separation studies with biphasic systems based on hydrophobic IL and water. However, it is necessary to warn that possible denaturation of biomacromolecules may occur due to the effect of IL. Faced with this issue, the aqueous two-phase system (ATPS) is composed of two liquid phases, which, although immiscible, have water as a major component in both phases, thus providing a mild environment adequate for the separation and purification of biological macromolecules, for example. However, as the authors of this contribution specify, despite previous studies highlighting possible important synergies of the corresponding combination of IL-based ATPSs, a broad study of their properties is required. Following a detailed analysis of the state of the art, the authors point out the urgent need for further basic physicochemical studies on the structure and properties of these types of systems and on phase separation mechanisms. Moreover, they highlight the need to establish rules that guide the selection of the most suitable ILs for both the effective separation of each type of analyte and that are also more suitable for the desired environmental preservation.

## 3. A Final Reflection

The eleven contributions comprising this Special Issue confirm the significant potential of thermodynamic tools in addressing the broad diversity of problems of both basic theoretical interest and practical applications. The contributions presented herein also offer broad perspectives that can serve to promote new research in fields of significant interest at present, such as process engineering, the field of human health, or environmental preservation.

Given the broad scope of its applications and the fact that, despite its maturity, there are still positive prospects for the development of the theory of thermodynamics, this subject must be maintained in the curricula of academic training centers and in research laboratories with full vigor. The current reformulations of scientific fields and their new interactions represent more than a mere threat to classical studies, a horizon of opportunities to which thermodynamics has much to contribute.
